# Testing Surrogacy Assumptions: Can Threatened and Endangered Plants Be Grouped by Biological Similarity and Abundances?

**DOI:** 10.1371/journal.pone.0051659

**Published:** 2012-12-11

**Authors:** Judy P. Che-Castaldo, Maile C. Neel

**Affiliations:** 1 Department of Plant Science and Landscape Architecture, University of Maryland, College Park, Maryland, United of America; 2 Department of Entomology, University of Maryland, College Park, Maryland, United of America; University of Kent, United Kingdom

## Abstract

There is renewed interest in implementing surrogate species approaches in conservation planning due to the large number of species in need of management but limited resources and data. One type of surrogate approach involves selection of one or a few species to represent a larger group of species requiring similar management actions, so that protection and persistence of the selected species would result in conservation of the group of species. However, among the criticisms of surrogate approaches is the need to test underlying assumptions, which remain rarely examined. In this study, we tested one of the fundamental assumptions underlying use of surrogate species in recovery planning: that there exist groups of threatened and endangered species that are sufficiently similar to warrant similar management or recovery criteria. Using a comprehensive database of all plant species listed under the U.S. Endangered Species Act and tree-based random forest analysis, we found no evidence of species groups based on a set of distributional and biological traits or by abundances and patterns of decline. Our results suggested that application of surrogate approaches for endangered species recovery would be unjustified. Thus, conservation planning focused on individual species and their patterns of decline will likely be required to recover listed species.

## Introduction

Policy makers and conservation managers strive to use the best available science to determine strategies for species conservation. For a vast majority of species, application of many scientific tools is limited because data for the species of interest are fragmentary, incomplete, or simply unavailable. To facilitate decision making in policy- and management-relevant time frames, scientists and practitioners have long sought indicators or surrogates to provide information about poorly known species to guide conservation and management [1]–[3]. Surrogate approaches lie between generic rules of thumb and detailed study of every species, and are appealing because they enable conservation of biological diversity or monitoring of ecosystem condition without comprehensive knowledge of every species or ecosystem element. They have been applied in a wide range of conservation situations including systematic reserve selection, forest management, and ecosystem management and monitoring (e.g. [4], [5], [6]).

In the broadest sense, surrogate approaches encompass all methods that apply principles from theory in ecology, population biology, and population genetics to determine conservation strategies in absence of species-specific information [2], [7], [8]. More typically a surrogate species approach is employed, in which information about one or more well-studied or representative species (“surrogate species”) is applied to one or more poorly known species of conservation concern (“target species”). Surrogate species may be chosen based on a range of biological similarities with target species. They may overlap with target species in terms of ecological requirements or geographical ranges (indicator and focal species; [2], [9]), control target species abundance through trophic interactions (keystone species; [10], [11]), have close phylogenetic relationships with targets (species groups; [12]), or have broad ecological requirements that encompass those of many species (umbrella species; [2], [13], [14]). Based on these biological relationships and similarities, benefits from protection or management of surrogates are inferred to extend to target species. Thus, use of surrogate species for conservation planning employs the assumption that species sharing biological traits or relationships will also be similar in terms of their distribution, abundance, or response to management.

An important potential application of surrogate approaches is in endangered species recovery planning. Most threatened and endangered species listed under the U.S. Endangered Species Act (ESA) lack sufficient data to directly assess extinction risk [15]. However, such assessment is necessary to help develop the objective and measureable recovery criteria that are required (ESA Sec. 4(f)(1)) to “bring any endangered species or threatened species to the point at which the measures provided pursuant to this Act are no longer necessary” (ESA Sec. 3(3)) and thus allow delisting of the species. These criteria usually consist of the number of populations or individuals needed to ensure species persistence, but can also include extent of habitat or range [15]. Severe lack of information for most species makes establishing defensible criteria challenging. As an alternative to direct assessments for all species, some researchers have attempted to estimate levels of extinction risk using biological traits or other surrogate characteristics [e.g.,16,17,18]. Extending this relationship, others have tested whether biologically similar species have similar recovery criteria, because the criteria to alleviate similar levels of extinction risk should also be similar [19]–[22]. Using traits to predict extinction risk assumes that species with similar traits share not only similar demographic rates and trajectories, but also other factors that determine species extinction (such as extrinsic threats).

Critics have long contended that surrogate approaches are ineffective because these underlying assumptions are likely unmet in most applications [23]–[27]. Further, in many cases the assumptions are not explicitly stated, and when they are stated they are rarely tested. As a result, the potential utility of surrogate approaches for most species is unknown. The few studies testing surrogacy assumptions for reserve selection have found no or weak correspondence between the presence, abundance, or richness of surrogates and those of the target taxa [28]–[30]. Selection of conservation sites based on one taxonomic group rarely represents other groups well and the degree of spatial overlap between groups is idiosyncratic [31]–[37].

Despite these criticisms, there is renewed interest in using surrogate species in conservation. With a need to address climate change in recovery planning but limited information on its impacts, surrogate approaches may be used to predict responses and future distributions of threatened and endangered species (e.g. [38]). Additionally, recent U.S. Fish and Wildlife Service guidance for Strategic Habitat Conservation (SHC) and Landscape Conservation Cooperatives outlined steps to implement the surrogate species approach in their conservation planning process [39]:

Because it is impractical and inefficient to conserve landscapes by considering requirements for all species present, selecting a subset of species to serve as surrogates for a broader array of biological outcomes is a practical first step and helps fulfill an important step in the biological planning component of SHC. As conservation practitioners, we will use these species to identify where on the landscape to target conservation efforts, what types of actions to take, and how much effort is needed.

Thus it is essential to continue testing assumptions to determine if there are circumstances in which the use of surrogates is appropriate [25]. In the case where a common or well-studied species is chosen to represent the demographic trends or management responses of a group of listed species, two specific assumptions must be met. First and fundamentally, there must be groups of threatened and endangered species that are sufficiently similar in multiple characteristics and/or threats to form identifiable groups that would justify representation by a surrogate species. Second, the groups of species must respond similarly to management and threat abatement as the surrogate species. In this study, we tested the first assumption by searching for groups of species with similar characteristics in the threatened and endangered plant species listed under the ESA. In addition to allowing potential representation by surrogates, groups of biologically similar listed species may share similar conservation needs and thus may be managed as a group to facilitate recovery planning. In this study, we used tree-based statistical models to examine whether listed plant species can be grouped based on a set of biological traits alone, their previous abundances and patterns of declines alone, or a combination of traits and abundances. This analysis will determine whether there are identifiable groups of listed species, and if so, identify traits that are important for defining these groups.

## Materials and Methods

### Variables Quantified

We compiled data on previous abundances and biological traits from recovery plans for the 642 listed plant species with final approved plans as of December 2009. We recorded the number of historically known populations, number of populations at listing, number of populations at plan writing, total number of individuals at listing, and total number of individuals at plan writing. To quantify the pattern of decline for each species, we calculated the proportion of historical populations remaining at plan writing and that at listing, the proportion of populations at time of listing remaining at plan writing, and the proportion of individuals at time of listing remaining at plan writing (Table 1).

**Table 1:** Summary of abundance variables included in our analyses.

	N	Minimum	Mean ± SD	Maximum
Number of populations				
Historical	408	1	16.9±35.48	475
At listing	415	0	7.2±13.74	173
At plan writing	601	0	11.0±21.63	231
Proportion of historical				
remaining at listing	287	0	0.68±0.278	1.0
Proportion of historical				
remaining at planwriting	406	0	0.67±0.272	1.0
Proportion at listing				
remaining at planwriting	395	0	1.00±0.398	7.8
Number of individuals				
At listing	380	0	7919±79910	1,500,000
At plan writing	478	0	240200±4579090	100,000,000
Proportion at listing				
remaining at planwriting	352	0	52.1±763.8	14290

We collected data on eight biological and distributional traits (hereafter referred to as “traits”): maximum plant height (m), maximum flower size (cm), life form (herb, lichen/moss, shrub, subshrub, tree, or vine), life history duration (annual, perennial, or short-lived perennial; some species fell in more than one category), reproductive mode (clonal, clonal and sexual, or sexual with no evidence of clonal reproduction), reproductive repetition (monocarpic or polycarpic), physiographic division [40] (Appalachian Highlands, Arctic, Atlantic Plain, Canadian Shield, Hawaii and Pacific Islands, Interior Highlands, Interior Plains, Intermontane Plateaus, Pacific Mountain System, Rocky Mountain System, West Indian; some species fell in more than one category, creating 23 distinct combinations of divisions), and range area (m^2^). We estimated range area as the area of intersection between the physiographic section [40] and the state(s) of occurrence listed in recovery plans, because actual range area is rarely provided in plans. We searched for numerous other traits but found the relevant data to be lacking for most species. Together, these traits represented the compromise between variables that have been found to relate to extinction risk and rarity and those that were available for a sufficient number of species to allow analysis. Taxonomic family contained too many levels (109 families) to be included as a predictor in the random forest analyses, and there were too few species per family (from n = 1 for Poaceae to n = 27 for Asteraceae) to allow analysis by family.

### Analyses

To examine whether listed plant species can be grouped based on similarities in traits, prior abundances representing patterns of declines, or a combination of traits and abundances, we used the ensemble classification and regression tree method random forests (RF) [41], [42]. Tree methods are especially useful for exploring large datasets that contain complex interactions between combinations of continuous, ordinal, and categorical variables. RF averages predictions over a set of trees built from bootstrap samples of the dataset, providing more robust predictions than single tree analyses [43]–[45]. In an uninformed RF the data are modeled without a response variable to assess whether there is inherent structure in the data. The original dataset is classified as group one and a second group of data is created through random permutation of the original data, and RF is used to re-assign the combined data into two groups based on predictor variables. If there is structure in the original data, RF will correctly reassign the same groups with error rate <50%. By convention, <40% error indicates significant grouping whereas higher error rates indicate random group assignment.

We used the *randomForest* function in the R package *randomForest* [46] to run RF. For each analysis we built 1000 trees with four randomly chosen predictor variables tried at each node (mtry = 4), except in the individual-based abundances model in which there were only three total predictors (mtry = 3). Different mtry values were tested and produced similar results (not shown). To assess model accuracy, we used the out-of-bag classification error (OOB error), which was the mean squared error calculated using only the observations that were not used to build the individual trees. RF calculates the importance of each variable as the mean increase in classification error when values for that variable are randomly permuted. That is, altering the values of an important variable would yield a large increase in error, whereas error will be less affected by permutation of a variable with little classification power. RF also calculates a proximity matrix consisting of the number of times each pair of observations are placed in the same terminal node, a measure of similarity between observations that can be used to visualize grouping structure in metric multidimensional scaling plots (using the *cmdscale* function in R [47]).

We performed three sets of analyses: classification of species based on traits alone, previous abundances alone, and both traits and abundances (Table 2). For the traits analyses, we ran an additional model including the year of recovery plan approval and listing status at plan writing (threatened or endangered) because they had previously been found to be important predictors of recovery criteria for birds [19]. For models including abundances as predictors, we also ran separate models including only population-based abundances or only individual-based abundances to examine whether the measure of abundance affected classification. Because all examined variables were missing data from at least one species, each analysis used a different subset of the data ranging from 70 to 352 species (Table 2).

**Table 2:** Summary of results from unsupervised random forest analyses examining whether threatened and endangered plant species can be grouped by distributional and biological traits, previous abundances, or a combination of traits and abundances.

Variable	Analysis							
	Traits only	Traits with status, plan year	Abundance only	Abundance - pop. only	Abundance - indiv. only	Abundance and traits	Abundance and traits - pop. only	Abundance and traits - indiv. only
N	213	213	197	283	352	70	96	130
Population abundances								
N Pop. Historical			✓	✓		✓	✓	
N Pop. Listing			✓	✓		✓	✓	
N Pop. Writing			✓	✓		✓	✓	
N Pop. Listing/Historical			✓	✓		✓	✓	
N Pop. Writing/Historical			✓	✓		✓	✓	
N Pop. Writing/Listing			✓	✓		✓	✓	
Individual abundances								
N Indiv. Listing			✓		✓	✓		✓
N Indiv. Writing			✓		✓*	✓		✓
N Indiv. Writing/Listing			✓		✓	✓		✓
Traits								
Max. height	✓	✓				✓	✓	✓
Max. flower size	✓	✓				✓	✓	✓
Life form	✓	✓				✓	✓	✓
Duration	✓	✓				✓	✓	✓
Reproductive mode	✓	✓				✓	✓	✓
Reproductive repetition	✓	✓				✓	✓	✓
Physiographic division	✓	✓				✓	✓	✓
Range	✓	✓				✓	✓	✓
Status		✓						
Plan year		✓						
Out-of-bag error (%)	50.00	50.23	46.70	46.64	29.40	50.71	50.52	48.85

Check mark indicates the variable was included in an analysis, and asterisk indicates the variable was identified as an important grouping variable.

## Results

### Biological Traits as Grouping Variables

**Figure 1:**
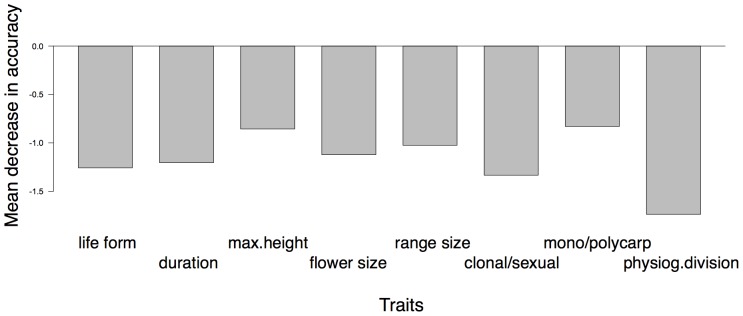
Traits variable importance values. Variable importance for the distributional and biological traits from the random forest analysis examining whether listed plant species can be grouped by traits only (n = 213). Variable importance is measured as the mean decrease in model classification accuracy when values for that variable are randomly permuted. Abbreviations: duration = life history duration, max.ht. = maximum plant height, max.flower = maximum flower size, range, reprod.mode = reproductive mode, reprod.repetition = reproductive repetition, physiogdiv = physiographic division.

We found no evidence of grouping among listed plant species based on distributional and biological traits: OOB error was 50.0% for the model including only traits and 50.2% for the model including traits, listing status, and plan year (Table 2). Variable importance values for all traits were negative (Fig. 1) including those for status and plan year (not shown), indicating that all examined traits were uninformative for classifying listed plant species. Because listing status and plan year did not appear to be important grouping variables and are not strictly species traits, we excluded them from further analyses.

### Previous Abundances as Grouping Variables

**Figure 2:**
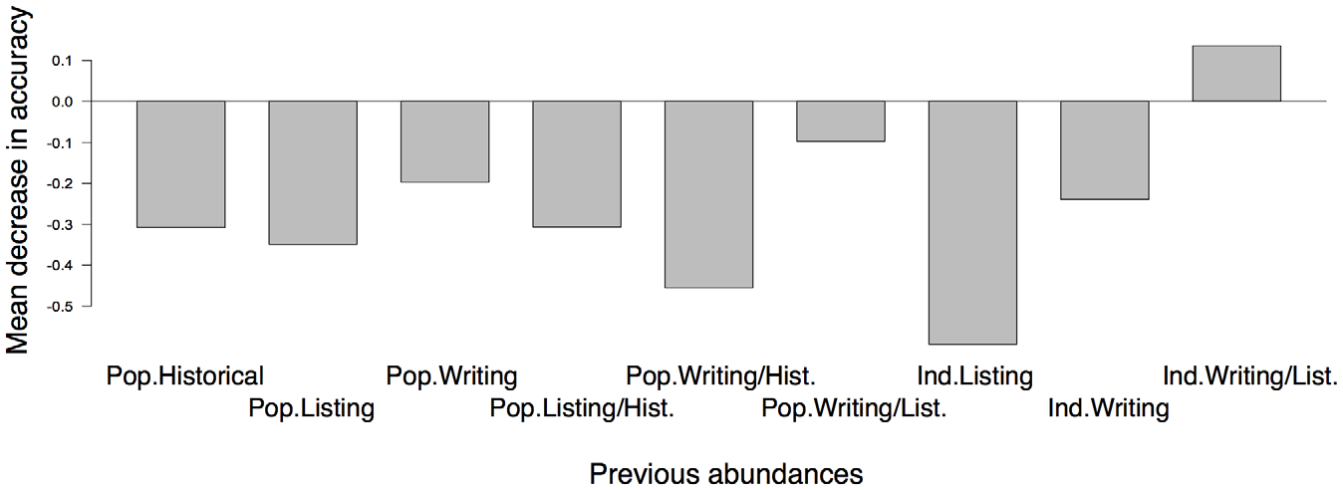
Abundance variable importance values. Variable importance for the previous abundance variables from the random forest analysis examining whether listed plant species can be grouped by previous abundances only, including both population-based and individual-based abundances (n = 197). Variable importance is measured as the mean decrease in model classification accuracy when values for that variable are randomly permuted. Abbreviations: Pop.Historical = Number of historical populations, Pop.Listing = Number of populations at time of ESA listing, Pop.Writing = Number of populations at time of recovery plan writing, Pop.Listing/Hist. = Proportion of historical populations remaining at time of listing, Pop.Writing/Hist. = Proportion of historical populations remaining at time of plan writing, Ind.Listing = Number of individuals at time of listing, Ind.Writing = Number of individuals at time of plan writing, Ind.Writing/List. = Number of individuals at time of plan writing remaining at time of listing.

**Figure 3:**
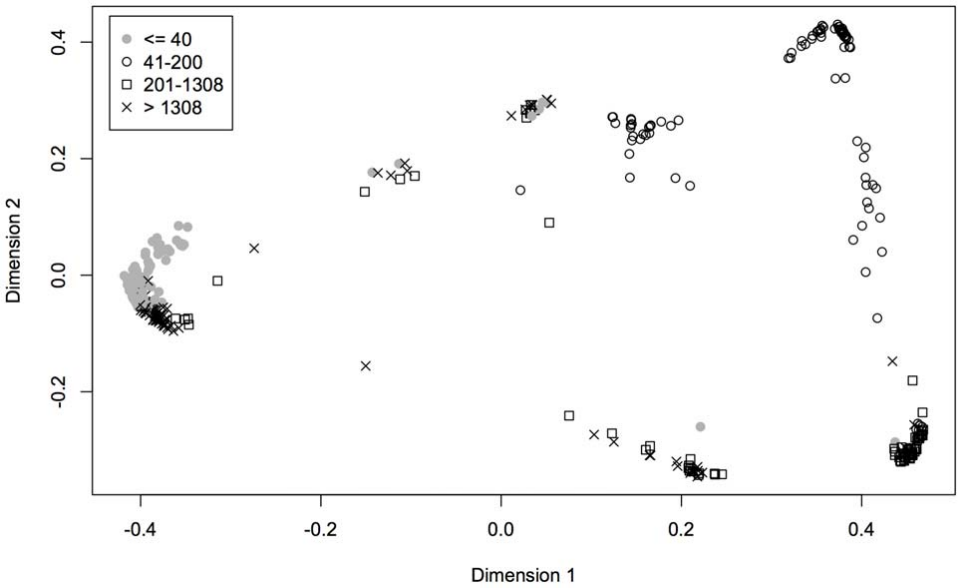
Grouping by number of individuals at plan writing. Multidimensional scaling plot visualizing in two dimensions a matrix of proximity scores from the random forest analysis examining whether listed plant species can be grouped by individual-based abundances only (n = 352). Proximity scores are the frequencies that two observations are placed in the same terminal node, and are a measure of similarity between species. Different symbols represent the four quartiles of the number of individuals at the time of plan writing (1^st^ quartile: ≤40 individuals, 2^nd^ quartile: 41–200 individuals, 3^rd^ quartile: 201–1308 individuals, 4^th^ quartile: >1308 individuals).

There was no evidence of grouping based on previous abundances when the analysis included both population-based and individual-based measures of abundance, and the OOB error was 46.7% (Table 2). Variable importance values for all abundance variables were negative or positive but small compared to the negative values for other variables (Fig. 2), again indicating low importance for classification. Results were similar when the analysis included only population-based abundances (Table 2). There was, however, significant grouping in the analysis including only individual-based abundances (OOB error = 29.4%; Table 2), and the number of individuals at plan writing had the highest variable importance value (mean decrease in model accuracy from variable permutation are -0.12, 0.06, and -0.06 for the number of individuals at time of listing, number of individuals at time of plan writing, and number of individuals at time of plan writing remaining at time of listing, respectively). To visualize this effect, we used multidimensional scaling to plot in two dimensions the matrix of proximity values from this analysis by the quartiles of the number of individuals at the time of plan writing (Fig. 3). This plot suggested there may be a grouping of species with ≤40 individuals at plan writing, but this group was not clearly separated from species with >1308 individuals at plan writing (Fig. 3). Species that had 41–200 individuals at plan writing also showed some tendency to group along Dimension 1, but they were not tightly aggregated on that axis and were even more dispersed on Dimension 2.

### Biological Traits and Previous Abundances as Grouping Variables

We found no evidence of grouping among listed plant species based on both traits and previous abundances: OOB error was >49% for all three models that included traits and either population-based abundances, individual-based abundances, or both (Table 2). Variable importance values for all trait and abundance variables were negative (not shown).

## Discussion

Our results demonstrated that ESA-listed threatened and endangered plant species cannot be grouped based on their biological traits or most of the abundance variables we examined. We did find that species with <40 individuals at the time of plan writing were similar in terms of their individual-based abundance measures. One possible explanation is that these species are rare in general (either naturally or as a result of decline) and therefore have low abundances at all time points measured (at listing, at plan writing, and proportion remaining at plan writing). Species that are more abundant, on the other hand, exhibit greater variation in these abundance measures and therefore are less similar. However, although the group of species with <40 individuals at plan writing was statistically significant, the grouping was not sufficiently well defined (Fig. 3) to justify group management based on these abundances alone.

The overall lack of grouping suggests low potential for widespread use of surrogacy to guide recovery planning because species do not meet the fundamental requirement of forming biologically similar groups. For the species we examined, using surrogates to define recovery criteria would be inefficient because a given surrogate could only be expected to represent a few at-risk species at best. The lack of similarity among listed species may indicate a true difference in terms of their intrinsic characteristics and patterns of decline, but it may also be due to data limitations even though we went to great lengths to develop a comprehensive database. For example, each of the traits examined had missing data for many of the species, and actual similarities may not have been illuminated due to insufficient data. However, managers tasked with choosing surrogate species will be faced with the same level of data deficiency given our data come from actual recovery plans.

The lack of grouping may also have resulted because listed species represent a subset of plant species that share similar values for the traits examined and cannot be further subdivided. For example, they all have relatively low abundances and have experienced declines, which are related to their threatened and endangered status. If their threatened status results in a small range of values for each trait represented in our dataset, there may not be sufficient variation among species to split them into more refined groups. It is possible that comparing non-listed and listed species would have presented a broader range of traits and enabled grouping species by traits. However, such groupings would not meet our goal of finding suites of listed species that can be managed similarly or that could have similar recovery goals. Further, although declines and abundances may be similar, listed plant species represent a broad range of plant life history characteristics and thus they are unlikely to lack variation based on these traits.

Previous studies testing the assumptions of surrogate approaches primarily examined how well abundance or distribution of surrogates predicted abundance or distribution of target species [28]–[37]. One of the few studies to test whether species sharing similar traits also share demographic characteristics (e.g., population abundance or decline) demonstrated that temperate birds with similar migratory status and the same feeding guild exhibited as much variance in abundance as all birds combined [48]. Although not specifically focused on surrogacy, studies attempting to link various life history traits to species rarity [49]–[51] or to extinction risk [16]–[18], [52]–[54] have also failed to yield consistent and predictable relationships. These results suggest that even if there were groupings of endangered species that shared biological traits, their demographic trends would likely not be determined by those traits alone and therefore would not be well predicted based on surrogacy.

Because relationships between surrogates and target species have been difficult to generalize, researchers recommend testing surrogate assumptions on a case-by-case basis [12], [26], [30], [55]. In other words, effective implementation of the surrogate approach requires monitoring the full set of target species in order to evaluate its success. Others suggest devoting resources to direct monitoring of target species rather than to surrogate approaches that require such extensive verification [25]. Based on previous studies and our current findings of failure to meet surrogacy assumptions, we agree that individual-species monitoring and recovery planning are likely required to develop defensible recovery criteria.

Currently, the only relatively standardized method to determine species-specific quantitative recovery criteria is the population viability framework based on minimum numbers of individuals required for a specified probability of persistence [56]. Vital rates estimated from demographic approaches can be used to determine whether populations are on trajectories toward persistence versus extinction [57] and structured population models can identify stages in the life cycle that are most important to population growth and the degree to which they are affected by stochasticity [58]. However, from a practical standpoint, use of population viability analyses (PVAs) for establishing recovery criteria and management actions is precluded for most species due to intensive information requirements. In fact, PVAs have only been used to help determine downlisting and delisting criteria for five listed plant species (in two recovery plans), and included in the description of basic natural history for only nine listed species [59]. It is possible that species with sufficient demographic data to develop PVAs may serve as surrogates for listed species if they are sufficiently similar biologically (e.g. [60]), but the extent of similarity between these groups of species is unknown. Further, biologically similar species in the same landscape do not have similar abundances [48], and vital rates are likely more variable. Finally, many consider the use of PVA to be inappropriate for setting absolute minimum numbers and suggest that it should only be used for comparative risk analysis [61]–[64].

Development of science-based recovery criteria and management actions for each listed species does not necessarily need to involve a PVA or the intensive demographic data collection that PVAs require. Rather, the type and extent of monitoring needed and the resulting management strategy should depend on the type of decline that a species has experienced. In general there are three primary types of declines that threaten species persistence: decreases in numbers of individuals within populations, range reduction without loss of populations, and loss of whole populations (which may occur with or without range reduction) [15], [65]. Each type of loss has different effects on species persistence and thus requires different management actions for recovery [66]. Species are often threatened by more than one type of decline, and the relative magnitude of each type of decline can be used to prioritize conservation actions [65].

Species that experience declines in population size have reduced population densities and can be affected by problems associated with small population sizes, such as decreased genetic diversity and susceptibility to stochastic events. In this case managers should prioritize threat reduction that impacts local population size or vital rates because the intact habitat range could still be sufficient to support species recovery when threats are removed. When range has been lost but populations remain, habitat restoration and protection would be required in addition to threat abatement. Managers may also need to establish connections among isolated patches, especially when dispersal abilities are limited, in order to avoid isolation and to facilitate gene flow and recolonization. Finally, when entire populations are lost, species can suffer from either range contraction, increased isolation, or both. This type of loss is more common in plants than in animals [65], and perhaps as a result has received less attention in terms of its conservation implications than other types of decline. Species that have lost populations may require spatially strategic reintroduction efforts combined with habitat protection and threat abatement to enable recovery.

In short, we are concerned that surrogate approaches and similar shortcuts are not supported by the best available science and further preclude the understanding of the status and trends of listed species. Although the prospect of determining science-based, quantitative recovery criteria and management actions for every listed species is a daunting task, focusing on the types of decline and their relative magnitudes that result from threatening processes may improve the efficiency of the process. Currently it is not possible to distinguish between the types of decline for all listed species or which type has had the greatest impact on species survival based on information provided in recovery plans. This is because historical range extents are often unknown or have not been explicitly documented, and most plans only describe declines qualitatively [65]. However, such information would enable further analyses of whether recovery criteria are consistent and objective. Moreover, the patterns of decline are likely related to the specific threats that have caused the decline. The type and severity of the various threatening processes contributing to species extinction also need to be more specifically quantified in recovery plans.
